# The common point for forensic and anthropologic genetics and individualized medicine: Ninth ISABS Conference on Forensic and Anthropologic Genetics and Mayo Clinic Lectures on Individualized Medicine, Bol, Croatia, June 22-26, 2015

**DOI:** 10.3325/cmj.2015.56.177

**Published:** 2015-06

**Authors:** Manfred Kayser, Tamas Ordog

**Affiliations:** 1 Department of Forensic Molecular Biology, Erasmus MC University Medical Center Rotterdam, Rotterdam, the Netherlands *m.kayser@erasmusmc.nl*; 2 Center for Individualized Medicine, Mayo Clinic, Rochester, MN, USA *Ordog.Tamas@mayo.edu*

This issue of the *Croatian Medical Journal,* the official journal of the International Society for Applied Biological Sciences (ISABS), is the tenth special issue over the past eighteen years containing articles prepared for the biennial ISABS conference.

Over the years, ISABS meetings have developed into a unique conference series highlighting advances in three major areas of applied genetics. Although “interdisciplinary” has become an *epitheton ornans* of modern life sciences, most scientific conferences are in fact dedicated to specialty or subspecialty areas. ISABS breaks this mold by bringing together forensic, anthropological, and medical geneticists for a five-day celebration of the transformation of their respective fields. By providing a venue for scientists and students from different fields united in their effort to apply molecular genetic principles and techniques to societally relevant issues, the Ninth ISABS Conference aims not only to facilitate discussion of the latest scientific achievements within these three areas of applied genetics, but also to cross-fertilize these fields. This year’s conference will further stimulate the interaction among scientists from the three disciplines by avoiding parallel sessions. We hope that the participants will appreciate and use this opportunity to attend all sessions regardless of their specific expertise.

Recognizing the breadth of topics included in the Ninth ISABS Conference and the need to pass the baton to the new generation of leaders, the founding organizers Dragan Primorac (Split and Osijek, Croatia), Moses Schanfield (Washington DC, USA), and Stanimir Vuk-Pavlović (Rochester, Minnesota, USA) tasked two Program Directors with designing the scientific programs in forensic genetics, anthropological genetics, and medical genetics.

The introductory session highlights the three major conference topics by lectures on the thirty-year history of forensic DNA analysis, genomics, and individualized medicine, the recent revolution of ancient DNA analysis, and advances in the application of glycobiology to individualized medicine. The keynote address by Anthony Atala (Winston-Salem, North Carolina, USA), a pioneer in the field, is devoted to regenerative medicine.

Manfred Kayser put together six thematic half-day sessions of forensic and anthropological genetics focusing on the most recent developments. The three forensic genetic sessions are devoted to DNA investigative intelligence, next generation DNA sequencing in forensics, and advances in routine forensic DNA analysis, and the three anthropological genetic sessions to the analysis of ancient human genomes, modern human genomes, and human genetic history of the continents. Both programs are driven by recent achievements in massively parallel DNA sequencing (MPS), also referred to as next generation sequencing. MPS has revolutionized anthropological genetics in ancient DNA analysis and holds great promise for population-based anthropological genetics using contemporary samples as well as for forensic genetics, especially if costs can be further reduced. Another recent addition to forensic genetics is the use of DNA and RNA for investigative purposes, a topic to which a conference session is dedicated. Such information allows linking (or excluding a link between) genetically identified sample donors and criminal acts, or leads police investigation to find unknown perpetrators unidentifiable by conventional DNA profiling; hence, it broadens the forensic use of genetics beyond the courtroom.

Tamas Ordog organized the medical genetics program, the Mayo Clinic Lectures on Individualized Medicine. The lectures start with an overview of the rapidly expanding use of whole-exome and transcriptome analysis via MPS in individualized patient care. The two half-day sessions include talks on host, microbial, and cancer genetics as well as epigenomics, a rapidly emerging discipline making its way into medical diagnostics and therapy. Recognizing the promise of epigenomics in diagnosis and therapy of cancer and functional and degenerative diseases, emphasized is the role of epigenetic changes in carcinogenesis, epigenetic biomarkers for early, noninvasive detection of cancer and neurodegenerative diseases, and the application of multi-parameter epigenomic testing to individualized medicine. The latter presentation includes the discussion of marker selection, logistics, bioinformatic analyses, as well as current barriers to incorporating epigenomic testing into clinical diagnostic arsenal. Talks on host genomics and epigenomics are complemented by a presentation on the role of microbiome in human physiology and disease. The program of the Ninth ISABS Conference will end with a lecture on twins ‘omics’ and personalized medicine.

Similarly to past ISABS meetings, the conference includes special evening events such as the lecture by the Nobel laureate Robert Huber (Martinsried, Germany), talks on “celebrity genetics”, poster sessions, Meet-the-Professor sessions, and social events. Meet-the-Professor sessions have been a unique feature of ISABS conferences as they stimulate the interaction of students and other participants with the invited experts in a more relaxed and informal atmosphere than typically available in more conventional settings.

In the partnership with PAIN-OMICS, a multidisciplinary consortium of leading clinical, academic and SME researchers in pain and different “omics” technologies, ISABS organizes a session that precedes the Ninth Conference.

Before the Ninth ISABS Conference (June 14-21, 2015), the American Academy of Forensic Sciences (AAFS) holds its 2015 International Educational Outreach Program in Croatia. The Memorandum of Understanding between AAFS and ISABS will be signed during the official ceremony held at the Ministry of Science, Education and Sports of the Republic of Croatia. For the first time in the history of ISABS, a joint ISABS-AAFS forensic science panel will be organized during the Ninth ISABS Conference.

Finally, the program directors and conference organizers express their gratitude to sponsors, academic and commercial, for the continuing support of this truly interdisciplinary, international conference. Bringing together a diverse group of internationally renowned scientists to facilitate research, collaborative endeavors, and education of the next generation of scientists in spite of adverse economic conditions in Croatia (and elsewhere) is a remarkable accomplishment that speaks volumes about the dedication of everyone involved, particularly the local organizers. We hope this tradition continues uninterrupted in the future, for the benefit of all.

